# Cardiac Fibroblasts and Cardiac Fibrosis: Precise Role of Exosomes

**DOI:** 10.3389/fcell.2019.00318

**Published:** 2019-12-04

**Authors:** Prabhat Ranjan, Rajesh Kumari, Suresh Kumar Verma

**Affiliations:** ^1^Division of Cardiovascular Disease, The University of Alabama at Birmingham, Birmingham, AL, United States; ^2^Department of Biomedical Engineering, The University of Alabama at Birmingham, Birmingham, AL, United States

**Keywords:** heart failure, paracrine signaling, exosome, miR, fibrosis

## Abstract

Exosomes are a group of extracellular microvesicles that deliver biologically active RNAs, proteins, lipids and other signaling molecules to recipient cells. Classically, exosomes act as a vehicle by which cells or organs communicate with each other to maintain cellular/tissue homeostasis and to respond to pathological stress. Most multicellular systems, including the cardiovascular system, use exosomes for intercellular communication. In heart, endogenous exosomes from cardiac cells or stem cells aid in regulation of cell survival, cell proliferation and cell death; and thus tightly regulate cardiac biology and repair processes. Pathological stimulus in heart alters secretion and molecular composition of exosomes, thus influencing the above processes. The past decade has yielded increasing interest in the role of exosomes in the cardiovascular system and significant contribution of cardiac fibroblast (CF) and mediated cardiac fibrosis in heart failure, in this review we had overviewed the relevant literatures about fibroblast exosomes, its effect in the cardiovascular biology and its impact on cardiovascular disease (CVD). This review briefly describes the communication between fibroblasts and other cardiac cells via exosomes, the influence of such on myocardial fibrosis and remodeling, and the possibilities to use exosomes as biomarkers for acute and chronic heart diseases.

## Introduction

**FIGURE 1:**
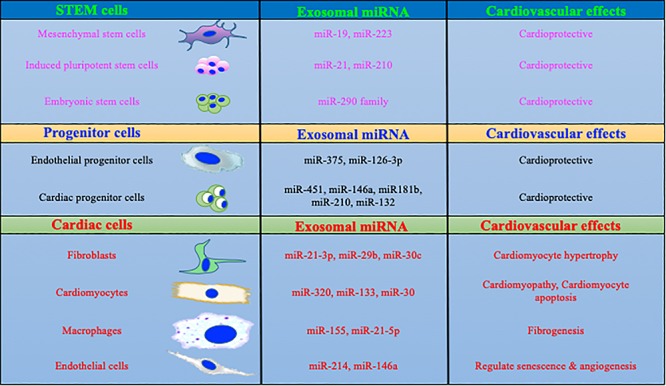
MicroRNAs packaged in exosomes regulate cardiac biology. Exosomal content is dependent on the parent cells and its physiological status. More specifically, exosomes derived from macrophages and fibroblasts are enriched in miRs which are involved with profibrotic and inflammatory signaling. In contrast, stem/progenitor cells derived exosome contains cardio protective miRs.

Intracellular communication is important in proficient and appropriate organization and function of various cells in multicellular organs. Multiple fundamental mechanisms are involved in the interactions between cells or even between different organs. For example growth factors, chemokines, adiponectin, small peptides, ECM proteins or sometimes direct cell-cell interaction are important for cellular communications (Corrado et al., 2013). However, in the last decade a considerable amount of experimental evidence has suggested that cells use a sophisticated method of communication using microvesicles called exosomes (Corrado et al., 2013; Maia et al., 2018). Exosomes are 30–120 nm size nanovesicles and have been identified in multiple cell types including stem cells for efficient intracellular communications (Mathivanan et al., 2010). Promising literature has shown that exosomes play a critical role in the shuttling of extraordinary sets of bioactive and signaling molecules which include membrane receptors, genetic materials, enzymes, cytokines and different bioactive materials in cells (Corrado et al., 2013; Cerezo-Magana et al., 2019). Thorough knowledge of a critical role for exosomes in the cardiovascular system is still developing, but establishment of novel tools and techniques in the past decade have boosted this research area significantly. Seminal work from others and our group has suggested that exosome-mediated intracellular signaling plays an important role in stem cell-mediated cardiac protection both in ischemic and hypertrophic heart failure (Sahoo et al., 2011; Mackie et al., 2012; Khan et al., 2015; Tseliou et al., 2015; Garikipati et al., 2018). Exosomes derived from stem cells provide an excellent cell-free system to improve cardiac function without significant immune response. Furthermore, cardioprotective factors such as miRs (Figure 1) and proteins packaged in stem cell exosomes may enhance the regenerative potential of stem cells to improve the endogenous repair process. Recently, it was shown that exosomes derived from IL-10-depleted EPCs exhibit altered exosomal content, which ultimately impairs the EPC’s cardiac repair property (Garikipati et al., 2017). Interestingly, modulation of miR-375 using a miRNA antagomir in IL-10KO exosomes partially rescued endothelial cell function (Yue et al., 2017). These studies clearly indicate that the direct role of exosomes in CVDs and repair processes and alterations in exosomal contents could be beneficial in the treatment of heart disease.

Numerically, heart consists mainly of CFs (Zhou and Pu, 2016) and during ischemic/hypertrophic insults these fibroblasts become activated and involved in cardiac fibrosis and remodeling (Travers et al., 2016). Bang et al. (2014) have shown that fibroblast-derived exosomes have the ability to enhance cardiac myocytes hypertrophy in pressure-overloaded myocardium. The constituent analysis of these exosomes indicates that they are rich in passenger strands of miR such as miR-21^∗^, a major signaling molecule which leads the hypertrophic signaling in heart. Interestingly, inhibition of miR-21 significantly reduced the cardiac hypertrophy and remodeling in this study (Bang et al., 2014). Furthermore, elevated level of miR-155 was found in macrophage-derived exosomes during heart injury (Wang et al., 2017). Intriguingly, Wang et al. (2017) has suggested that miR-155 in macrophage exosomes has potential to enhance proliferation and differentiation of resident fibroblasts and further exacerbate inflammation. These findings suggest that targeting selective molecules in cardiac fibroblast-derived (CF)-exosomes or inhibition of exosome secretion could be potential therapeutic approaches in heart failure treatment. It is also possible that exosomes from other cells such as immune cells can stimulate transition of naïve fibroblasts to activated myofibroblasts. Very limited literature is available regarding the activated fibroblasts exosomes and exosome-mediated paracrine signaling in cardiac fibrosis and remodeling. We hope that future rigorous studies on CF exosomes and mediated intercellular communications in the heart (between CFs and other cells or vice versa) will provide better understanding to develop novel therapies for CVDs. In this review article, we explore the current understanding of CFs; cardiac fibrosis; exosomes; exosomal biogenesis, structure, composition and involvement in cardiac fibrosis during heart failure. Additionally, we will discuss possibilities of exosomes as biomarkers for cardiac fibrosis and remodeling.

## Cardiac Fibroblasts and Cardiac Fibrosis

Excessive cardiac fibrosis is a major problem in nearly all types of heart disease and significantly attributed by activation and excessive proliferation of CFs (Ali et al., 2014; Travers et al., 2016). During development, the CF population changes dramatically and regulates cardiomyocyte proliferation through multiple signaling pathways (Banerjee et al., 2007; Ieda et al., 2009). However, during disease states, excessive ECM proteins such as collagen accumulate and expand in the cardiac interstitium, which disrupts heart contractile capacity and impairs its systolic and diastolic function (Janicki and Brower, 2002; Berk et al., 2007; Kong et al., 2013). Upon cardiac insult, for example acute MI, cardiomyocytes die and a massive inflammatory and fibrogenic responses are triggered to develop a fibrotic scar as a reparative response (Frangogiannis, 2012; Humeres and Frangogiannis, 2019). Activated CFs, termed myofibroblasts, are phenotypically modified cells with differential expression of excessive ECM proteins including collagens, MMPs, and their inhibitors (Cleutjens et al., 1995; Kawaguchi et al., 2011). As compared to CFs, myofibroblasts are more contractile and express significantly more α-SMA and periostin. At an early stage of cardiac stress, these changes contribute to an adaptive repair process but eventually lead to adverse cardiac remodeling and progression toward heart failure (Tomasek et al., 2002; Hinz, 2007, 2010; Shinde et al., 2017). With or without cardiac injury, aging, chronic kidney disease, diabetes and obesity may also trigger cardiac fibrosis and remodeling (Biernacka and Frangogiannis, 2011; Cavalera et al., 2014; Hayer et al., 2018). The homeostasis of collagen turnover is tightly regulated by CFs and any imbalance in collagen metabolism leads to cardiac fibrosis (Janicki and Brower, 2002; Rathod et al., 2016). Cardiac fibrosis is mainly categorized into four types, based on the location and cause. The most prevalent two forms are the reactive interstitial and the replacement fibrosis. Interstitial fibrosis mainly describes the expansion of endomysium and perimysium, is caused by progressive deposition of extracellular proteins in the interstitial space, and leads to cardiomyocyte death. Whereas, replacement fibrosis occurs by necrosis of cardiomyocytes and is associated with systolic ventricular dysfunction, hypertrophic CM and myocarditis (Hashimura et al., 2017; Liu et al., 2017; Frangogiannis, 2019). A third category is infiltrative interstitial fibrosis, which occurs due to infiltration of inflammatory cells in right ventricles of systemic sclerosis-associated pulmonary arterial hypertension (Overbeek et al., 2010). The fourth type, termed endomyocardial fibrosis, is a primary cause of congestive heart failure in children under 2 years of age and underlying causes for this type are not well established but include infections, autoimmunity, genetic factors, and nutritional deficiencies etc. (Rohit et al., 2013; Duraes et al., 2019). The pathophysiology of cardiac fibrosis is mostly attributed to excessive synthesis and accumulation of ECM proteins by activated myofibroblasts. Even though cardiac fibrosis is involved in most forms of CVD, clinical interventions targeting cardiac fibrosis are not yet in hand. Cell population heterogeneity and lack of identification of cell-specific markers add to the complexity of designing and improving therapeutic intervention to reduce cardiac fibrosis.

### Origin and Activation of Fibroblasts

**FIGURE 2:**
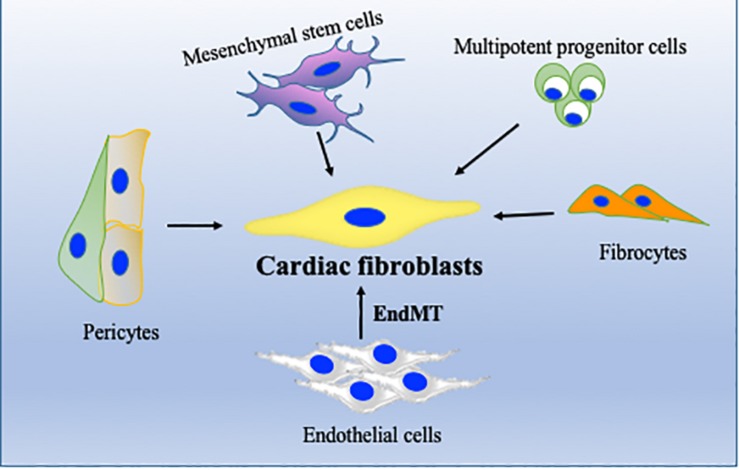
Cellular sources of cardiac fibroblasts (CFs). In addition to resident fibroblasts, CF can originate from endothelial cells, through endothelial-to-mesenchymal transition (EndMT), from bone marrow stem/progenitor cells, from perivascular cells or from fibrocytes. Their activation and differentiation to become activated fibroblasts/myofibroblasts are highly dependent on pathological stress on the heart.

Regardless of etiology, the origin of myofibroblasts remains controversial (Kong et al., 2014). Recent studies with lineage tracing strategies have suggested that cardiac fibrosis is primarily mediated by resident fibroblasts; however, other cell types (Figure 2) including monocytes/macrophages, endothelial cells, and hematopoietic fibroblast progenitors may also contribute to pathological fibrosis in heart (Ali et al., 2014; Kong et al., 2014; Moore-Morris et al., 2014, 2018). Endothelial cells and α-SMA-expressing mesenchymal cells have been shown to significantly contribute to fibrosis via their canonical Wnt-mediated endothelial-to-mesenchymal transition (EndMT) (Aisagbonhi et al., 2011). We have recently shown that bone marrow cells migrate to heart and transdifferentiate into myofibroblasts after myocardial damage, and thus contribute to tissue remodeling. In our study, we found that inflammatory stimulus acts as a catalyst for enhanced mobilization and homing of these bone marrow progenitor cells (Verma et al., 2017). In heart, cardiomyocytes/fibroblasts/resident macrophages secrete several chemokines such as SDF-1 which play an important role in migration of these cells to the heart (Mollmann et al., 2006; Chu et al., 2010). In addition to bone marrow cells, cardiac endothelial cells may also become myofibroblast-like cells by a process called EndMT and may be involved in pathological fibrosis during hypertrophic heart failure (Zeisberg et al., 2007).

### Stimulator of Cardiac Fibrosis

**FIGURE 3:**
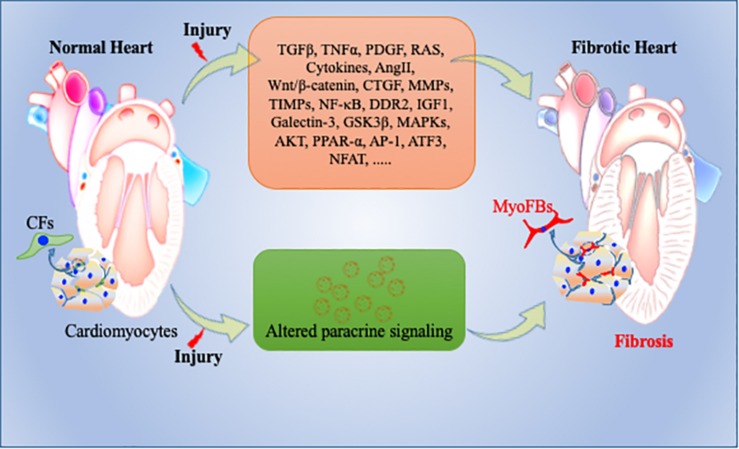
Pathophysiology of cardiac fibrosis. During normal remodeling, adequate ECM proteins secreted by cardiac fibroblasts (CFs) are important to maintain cellular integrity in heart. However, upon cardiac injury, CFs transdifferentiate into activated myofibroblasts, secrete excessive amounts of ECM proteins, and expand the cardiac interstitium as a wound healing process. Many key molecular determents are involved in this repair process including exosomes. Altered molecular and paracrine signaling pathways can contribute to exacerbated activation and *trans-*differentiation of fibroblasts to myofibroblasts and to adverse/pathological cardiac remodeling and heart failure. CFs = Cardiac fibroblasts; myoFBs: myofibroblasts.

Inflammatory stimulus and cardiomyocyte death are often the initial factors which stimulate a profibrotic signaling cascade in resident fibroblasts which secrete excess ECM proteins (Tomasek et al., 2002). The potential mechanism and type of profibrotic stimulation which leads this process mostly depends on the type of cardiac injury. Many different growth factors (TNFα, PDGFs, TGFβ), cytokines (IL-1, IL-10, IL-11), renin angiotensin system (RAS), and microRs are key modulators in initiation and progression of cardiac fibrosis (Saxena et al., 2013; Thum, 2014; Frangogiannis, 2015, 2019; Tao H. et al., 2016; Verma et al., 2017). RAS and TGFβ signaling are perhaps the most studied among all fibrotic pathways. Regardless of the type of cardiac injury, components of RAS are mostly produced by macrophages and resident fibroblasts and ultimately stimulate cardiac fibrosis by TGFβ signaling pathway. Upon activation, TGFβ modulates cellular functions in various cells including cardiomyocytes and fibroblasts and promotes the myofibroblast phenotype via canonical and non-canonical pathways (Kong et al., 2014). TGFβ-independent activation of human fibroblasts to myofibroblasts has also been studied and is involved in cardiac remodeling (Baranyi et al., 2019). Other than these stimulators, the roles of metalloproteinases (MMPs), TIMPs, and NF-κB are also well established in mediating cardiac fibrosis (Creemers et al., 2001; Kumar et al., 2011; George et al., 2016). As myofibroblasts are a major source of excessive ECM production, in addition to fibroblasts, macrophages also play an important role in ECM production and remodeling. Upon cardiac injury, macrophages adopt a more fibrotic M2 phenotype which has reduced expression of inflammatory cytokines like TNFα and interleukin-6 (IL-6) and increased secretion of IL-10, IGF1, TGFβ, and Gal-3 that are really key for ECM remodeling (MacKinnon et al., 2008; Suthahar et al., 2017). Non-immune cells like cardiomyocytes and CFs secrete pro-inflammatory cytokines to which myofibroblasts mostly respond and leads to excessive cardiac fibrosis (Yamauchi-Takihara et al., 1995; Porter and Turner, 2009; Aoyagi and Matsui, 2011). In addition to these inflammatory and profibrotic factors, GSK3β, β-catenin, TGFβ/SMAD-4, Wnt/β-catenin, MAPKs, and AKT signaling molecules and pathways play pivotal roles in cardiac fibrosis by regulating ECM metabolism, cardiomyocyte survival and proliferation, and maintaining wound healing after cardiac injury (Deb, 2014; Ma et al., 2018; Singh et al., 2019; Umbarkar et al., 2019; Figure 3). Among intracellular pathways, oxidative stress is a key factor that enhances cardiac fibrosis by triggering fibroblast proliferation and fibrotic signaling in heart. ROS generation has a dual role in cardiac fibrosis as studies reported both matrix-degradation and matrix-synthesis effects. High ROS levels increase TGFβ production and enhance CFs proliferation; paradoxically, high ROS also stimulates MMPs which facilitate ECM degradation (Siwik et al., 2001; Purnomo et al., 2013). Intriguingly, in the last decade, the role of ncRNAs, such as microRNA (miRNA), circular RNA and lncRNA, have been explored extensively in cardiovascular research and are suggested as an important trigger for various cardiovascular events including cardiac fibrosis and remodeling. Among these, microRNs are the most extensively studied NcRNA and play a critical role in regulation of fibroblast proliferation and fibrosis (Thum and Condorelli, 2015; Piccoli et al., 2016). For example, miR-21 is expressed in all heart cells and well characterized as profibrotic miRNA. It targets the expression of sprouty homology 1 (SPRY1), PTEN, and TGFβ receptor III (Huang et al., 2015). In contrast, miR-29 has been shown to reduce fibrosis by down-regulating expression of ECM genes, though in other studies miR-29 expression decreased during heart failure (van Rooij et al., 2008). Other miRNAs like miR-126, miR-15, miR-499, miR-24, miR-378, miR-1, miR-133, miR-26, miR-22, miR-199, miR-23, and miR-208 have also been studied considerably in different heart failure models (Montgomery et al., 2011; Melman et al., 2014; Tijsen et al., 2014; Suresh Babu et al., 2016; Figure 3).

## Paracrine Signaling and Its Role in Cardiac Fibrosis

Recently, enormous efforts have been made to explore the involvement of paracrine signaling in heart pathophysiology (Anthony and Shiels, 2013; Gartz and Strande, 2018). EVs, as a means of paracrine signaling, are bilayer membrane-bound cargoes and include exosomes, microvesicles and apoptotic bodies. Exosomes are extensively studied paracrine factors and can deliver various functional modulators such as proteins, lipids, DNA, mRNA, and miRNA. These transporter vesicles regulate a plethora of functions like cell differentiation, proliferation, senescence, cell death, cell–cell communication, angiogenesis, recycling of membrane lipids and proteins, and immunomodulation (Janowska-Wieczorek et al., 2005; Valadi et al., 2007; El Andaloussi et al., 2013; Xu and Tahara, 2013; De Jong et al., 2014). In the following section, we will focus on recent advancements in exosome research, especially on fibroblast biology and its role in myocardial fibrosis.

### Exosomes: Structure and Function

**FIGURE 4:**
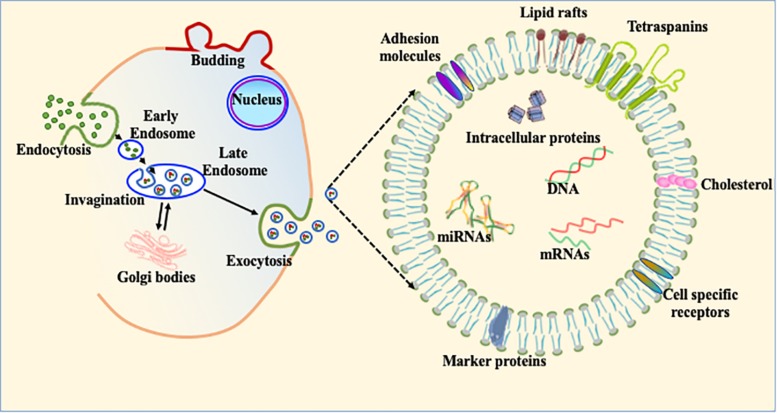
Exosome biogenesis and its constituents. The schematic diagram of exosomal biogenesis. Exosomes are membranous vesicles 30–120 nm in diameter formed by the inward budding of the cellular membrane. This can shed off inward and fuse with early endosomes which become known as late endosomes and multivesicular bodies (MVBs). These nanovesicles carry various cellular components, such as mRNA, microRNA, DNA, and proteins within their bilayer lipid membrane such as Alix, Tsg101, and tetraspanins.

Most living and actively functional cells secret 100–1,000 nm size micro-particles. Based on size, these microparticles are classified into three main subpopulations. The smallest particles (ranging from 30–120 nm) are termed exosomes (Figure 4). Exosomes were first discovered in 1946 as cellular waste and described as nanosized vesicles in 1981 (Chargaff and West, 1946; Valadi et al., 2007). Recent advances in biological science suggest that exosomes play important roles in multiple biological and pathological processes. Due to much complex biology and physiology in heart, exosome-mediated intracellular communication is an under-developed area in the cardiovascular field. Once exosomes are secreted into the extracellular system, they are stable for a relatively long period of time and can transfer cell specific signature signaling molecules to the target or recipient cells (Aryani and Denecke, 2016). Exosomes are mostly involved in cellular communication between different cell populations in multicellular organs. The main purpose of exosome-mediated intracellular signaling is to maintain cellular homeostasis and appropriate response to physiological stress. These nanovesicles contain various cellular components, such as mRNA, microRNA, DNA and membrane-bound or embedded proteins such as Alix, Tsg101 and tetraspanins (Frydrychowicz et al., 2015; Gurunathan et al., 2019). Tetraspanins are a group of transmembrane signaling proteins present in exosomes and in viable cells. The most common tetraspanins on exosomes are CD9, CD63, CD81, and CD82 and are commonly used as markers for characterization of exosomes. Exosomes may also contain Hsp (Hsp70 and Hsp90) and several intercellular adhesion molecules such as CD11a, CD11b, CD11c, CD18, CD146, CD166, and LFA-3/CD58 from their parent cells (Bobrie et al., 2011). In addition to these cellular proteins, exosomes contain a variety of genetic materials (mRNA and miRs) and are involved in angiogenesis, epigenetics, and gene regulation (Bobrie et al., 2011; Frydrychowicz et al., 2015). Although it was initially considered that exosomes are the entities whose primary function is to clear cellular waste, with increasing understanding of structural details and physiological function, study of cell-specific exosomes is currently a very hot area to explore disease pathobiology (Figure 4; Johnstone et al., 1987; Chang and Wang, 2019; Kelemen et al., 2019; Sole et al., 2019; Wang et al., 2019). Based on the broad array of content packaged in exosomes, we think that regulation of exosomal contents is a potential therapeutic strategy in heart disease treatment.

### Role of Exosomes in Cardiac Fibrosis

Emerging evidence suggests that exosomes are secreted by most cardiac, vascular and stem cells in heart (Sahoo et al., 2011; Mackie et al., 2012; Khan et al., 2015; Tseliou et al., 2015; Garikipati et al., 2018). Thus, we surmise that all the various cells in heart use exosomes to communicate with each other viz. cardiac myocytes to endothelial cells, endothelial cells to smooth muscle cells, fibroblasts to cardiac myocytes and vice versa to regulate physiological or pathophysiological processes (Hergenreider et al., 2012; Bang et al., 2014; Wang et al., 2014). Thus, any alteration either in signaling molecules packaged in exosomes or in exosomal machinery can affect physiological homeostasis which ultimately results in heart disease. Exosomes play a central role in many cardiac diseases including MI, hypertrophy and ischemia (Xu et al., 2017). Recent evidence has shown that cardiac cell communication via exosomes is altered during fibrosis, a key mediator for heart diseases (Cosme et al., 2017). These novel findings are driving active research to study the role of fibroblast-derived exosomes and the effects of exosomes from other cells on fibroblasts to understand the pathophysiological consequences in fibrosis and heart disease.

Normally CFs contribute ∼70% of the cardiac cells and support cardiomyocytes by producing ECM and by regulating proliferation and migration of other cardiac cells (Furtado et al., 2016). Thus, fibroblasts play an important role in cardiac repair. However, in certain circumstances excessive proliferation and differentiation of fibroblasts lead to fibrosis and heart failure. Recent literature has shown that exosomes may also modify cardiac repair and fate of fibrosis via modulation of fibroblast function (Barile et al., 2017; Wang et al., 2017). Exosomes derived from cardiac progenitor cells (CPC) have potential to activate naive fibroblasts to initiate the wound healing process for myocardial repair (Barile et al., 2017). During cardiac injury, activated macrophages use exosomes enriched in miR-155 to regulate fibroblast differentiation to myofibroblasts resulting in more fibrosis. Thus, macrophage-specific inhibition of miR-155 or direct inhibition of this miRNA could be potential therapeutic approaches for regulation of cardiac injury (Barile et al., 2017). Recently, Yang et al. (2018) have found that cardiomyocyte-derived exosomes can promote cardiac fibrosis via myocyte-fibroblast cross-talk. It has been shown that injured epithelial cells secrete exosomes enriched with profibrotic factors, which can lead to fibrosis (Tomasek et al., 2002; Lee and Kalluri, 2010; Borges et al., 2013). Recently, we have shown that EPCs from IL-10KO mice secrete exosomes which are enriched with profibrotic and antiangiogenic factors and miRs. Alteration in exosomal contents significantly reduced fibrotic signaling after exosome transplantation in heart post-MI (Garikipati et al., 2017; Yue et al., 2017). Thus, these publications indicate that exosomes from different cellular sources are acting as a significant determinant in regulating cardiac fibrosis. Though exosomes contain several important molecular signatures, the role of exosomal miRs and its significance in regulation of CVDs are recently highlighted in many articles (Valadi et al., 2007). We believe that modification of miRs using selective antigomir/mimic in exosomes can regulate pathological fibrosis and remodeling. To date, several miRs are identified which regulate fibroblast proliferation, differentiation and thus fibrosis (Garikipati et al., 2018; Table 1). Therefore, targeting those miRs in exosomes could be beneficial in reducing fibrosis and restoring heart function.

**TABLE 1:** Regulatory miRNAs associated with cardiac fibrosis.

**S. no.**	**miRNA**	**miRNA level**	**Cardiovascular disease**	**Target gene/pathway**	**Cardiovascular effects**	**References**
1	miR-433	Overexpression	Cardiac fibrosis	AZIN1 and JNK1	Induce CF	Tao L. et al., 2016
2	miR-21-5p	Overexpression	Left ventricular hypertrophy	PPARα	Induce Hypertrophy	Chuppa et al., 2018; Moghaddam et al., 2019
3	miR-21-5p, miR-135b	Overexpression	Left ventricular hypertrophy, Cardiomyopathy	Wnt and Hippo pathway	Induce Fibrosis	Zhang H. et al., 2016; Moghaddam et al., 2019
4	miR-22	Downregulation	Cardiac fibrosis	TGFβRI	Induce CF	Jazbutyte et al., 2013; Hong et al., 2016
5	miR-29	Downregulation	Cardiac fibrosis	TGFβ/BNP	Induce CF	van Rooij et al., 2008; Chaturvedi et al., 2015
6	miR-34a	Overexpression	Cardiac fibrosis after MI and IR injury	SMAD4	Induce CF	Huang et al., 2014
7	miR-208a	Overexpression	Cardiac fibrosis	Dyrk2	Induce CF	Shyu et al., 2015; Yang et al., 2018
8	miR-132	Overexpression	Cardiac fibrosis	PTEN gene, PI3K/Akt	Inhibit CF	Zhang et al., 2018
9	miR-29a-c	Downregulation	Cardiac fibrosis	TGF-β/Smad3	Induce CF	Roncarati et al., 2014; Zhang et al., 2014; Harmanci et al., 2017
10	miR-669a	Downregulation	Cardiac fibrosis	MyoD	Induce CF	Quattrocelli et al., 2013
11	miR-455	Overexpression	Cardiac fibrosis	CTGF, LncRNA H19	Inhibit CF	Chaturvedi et al., 2015; Huang et al., 2017
12	miR-155	Overexpression	Cardiac fibrosis	TGF-β1–Smad 2	Induce CF	Zhang D. et al., 2016; Wang et al., 2017
13	miR-425, miR-744	Downregulation	Cardiac fibrosis	TGFB1 3′UTR	Induce CF	Wang et al., 2018

### miR-Enriched Exosomes and Their Role in Cardiac Fibrosis

MicroRNAs (miRNAs, miRs) are highly conserved 21–25-nucleotide small NcRNA. They regulate target gene expression by binding to mRNAs and regulating the translation process (Thum and Condorelli, 2015; Piccoli et al., 2016). miRNA expression is altered in many CVDs including cardiac fibrosis and remodeling (Li et al., 2013; Tao L. et al., 2016). Recent advancements in technology helped to us to understand the direct role of miRs in cardiac biology and functions. For instance, during ischemic heart diseases, miRs play important roles in cardiac myocyte survival and thus improve heart function. Few miRs are exclusively expressed in muscle tissue (such as cardiac myocytes and skeletal muscles) and involved in cardiomyocyte contractility, survival and function. These miRs are termed myomiR (such as miR-1, miR-133, miR-206, miR-208, miR-486, and miR-499) (McCarthy, 2011; Chistiakov et al., 2016). As myomiRs are mainly expressed in muscle cells and play important roles in cell function, during myocardial damage, myomiR expression is altered tremendously (Gidlof et al., 2011). Interestingly, altered expression of many miRs such as miR-15, miR-21, miR-208a, miR-195, miR-29a, and miR-497 has potential to impair cardiac function post-injury (Hydbring and Badalian-Very, 2013; Porrello et al., 2013; Lin et al., 2015). Previous studies have suggested that CF activation and cardiac fibrosis are tightly regulated by sets of miRs termed fibrosis-associated miRNAs (Please see Table 1). Any alteration in these specific miRs can lead to exaggerated fibrosis. Interestingly, miR-433 is highly increased during myocardial ischemia and leads to cardiac fibrosis. This specific miR regulates MAPK 1 and TGF-β signaling pathways and thus enhances profibrotic signaling (Tao L. et al., 2016). Recently, Moghaddam et al. (2019) have mentioned that many miRs (such as miR-21, miR-22, and miR-24) are highly upregulated during acute ischemic injury. In addition, this group has also mentioned that miR-15, miR-34, miR-130, and miR-378 expression are noticeably reduced and are mainly responsible for the cardiac fibrosis after acute MI and IR injury models (Moghaddam et al., 2019). In summary, miRs from different cellular sources (Please see Table 1) can have ability to alter multiple molecular and cellular processes including cardiac fibrosis. Some sets of miRs can induce fibrosis and others can reduce it. Therefore, a balanced expression of these miRNAs is critically important during appropriate cardiac healing processes after any type of cardiac injury.

As we discussed, exosomes contain an extensive repertoire of genetic material including miRs. Recent reports have suggested that cells can also exchange miRs via exosomes, which can significantly alter the recipient cell’s biology and function (Guay et al., 2015; Khalyfa et al., 2016; Qiao et al., 2019). We have previously shown that miR-125b is an important miR in heart and plays an important role in activation of fibroblasts (Nagpal et al., 2016). Recently, Yang et al. (2018) found that exosomes derived from cardiomyocytes are enriched with miR-208a. At the molecular level, this study suggests that miR-208a enhances NFAT phosphorylation by targeting Dyrk2, preventing its entry into the nucleus in CFs, and therefore triggering fibrosis. Furthermore, Chaturvedi et al. (2015) have demonstrated that during exercise miRNA-29b and miRNA-455-enriched exosomes from cardiomyocytes can prevent fibrosis by downregulating MMP9 levels in diabetic mice. Furthermore, Bang et al. (2014) reported that in CFs, exosomal miRs are enriched with many miR passenger strands. In this study they found that fibroblast exosomes are enriched with miR-21^∗^ which has potential to induce cardiomyocyte hypertrophy by silencing expression of SORBS2 or PDLIM5 (Bang et al., 2014). Furthermore, Ang II-induced cardiac hypertrophy was effectively controlled by miR-21^∗^ inhibition in mice (Bang et al., 2014). In a very similar study, Lyu et al. (2015), showed that activated CF exosomes enhanced RAS signaling in cardiomyocytes, whereas inhibition of CF-exosome secretion by GW4869 (a potent EV inhibitor) significantly reversed Ang II-induced cardiac hypertrophy and remodeling. In addition, Wang et al. (2017) demonstrated that exosomal miR-155 inhibits both SOS and Suppressor of Cytokine Signaling 1 (SCS) expression, respectively, in fibroblasts and macrophages, thus regulating their proliferation. These studies suggest exosomes play important roles in fibroblast-mediated paracrine signaling. Furthermore, regulation of exosome biogenesis or content using pharmacological or molecular approaches could provide valuable therapeutic tools in regulation of heart failure.

### Exosomes May Act as a Potential Biomarker in Cardiac Fibrosis

Recently, attempts have been made to use miRNAs or other signaling molecules in serum or plasma as diagnostic biomarkers for cancer. Researchers have found that the molecular constituents of exosomes are highly associated with parent cell phenotype and concurrent physiological/pathological condition. Thus, we can believe that exosomes are replicas of parent cells in regard to their molecular constituents. During biogenesis, exosomes receive multiple proteins via processing through endosomal pathways. These proteins are displayed on the exosome surface and include, but are not limited to, tetraspanins, heat shock proteins (HSP70) and proteins from the Rab family, Tsg101 and Alix (Bobrie et al., 2011; van der Pol et al., 2012). Therefore, proteins on the exosome surface may be utilized as diagnostic tools, as they have been proven very specific and clinically relevant (Lin et al., 2015). It has been shown that body fluids are rich in exosomes, and the specific biomolecules inside of, or on the surface of, exosomes can act as a biomedical tool to determine the disease stage or progression (Takata et al., 2008; Street et al., 2011). Careful investigation of exosomes in body fluids of patients with high risk factors for CVDs may provide us clinically useful information to diagnose these diseases at much earlier stages than previously possible. For example, cardiomyocytes secrete various muscle-specific miRNAs through exosomes. During cardiac injury, elevated levels of these miRNAs can be detected in blood exosomes much earlier than detection of cardiac troponins or other markers (Xu et al., 2019). Enhanced levels of fibrosis-associated miRNAs (Table 1) such as miR-21, miR-425, miR-744, miR-208a, and others in plasma exosomes can also act as biomarkers in early diagnosis of hypertrophic heart diseases (Wang et al., 2018). In coronary bypass, patients’ plasma exosomes are enriched with miR-1 and miR-133 and thus these miRs can be used to indicate disease progress (Emanueli et al., 2016). Matsumoto et al. (2013) has suggested that microRNAs miR-34a, miR-192, and miR-194 can be used as biomarkers to determine heart failure as well. Most studies mentioned here, and many more, have clearly indicated that miRNAs or other molecules packaged in exosomes may act as prognostic markers for heart diseases. However, rigorous investigations must be carried out in large cohorts of human patients before reaching at any final conclusion. We are optimistic that, in the near future, exosomes will be a powerful diagnostic marker to determine the progress of heart disease at early stages and will help our fellow clinicians manage this deadly disease in a more efficient manner.

## Concluding and Prospective Remarks

Influence of exosome-mediated cardiovascular signaling and its role in CVDs have been rigorously studied and many insightful studies have been conducted and published in the recent past; however, we are still far from developing the exosome-based therapeutic for the treatment of CVDs. More in-depth research is warranted for fully understanding the biological aspects of loading, targeting, and delivery of exosomes, and for identifying the endogenous content of exosomes. Several unanswered questions remain to be addressed, such as (1) What regulates exosome biogenesis during heart failure? (2) How do cell type-specific exosomes exert their effect at the time of heart injury? (3) Does intense fibrotic response alter exosome-mediated signaling during CVD? (4) Are there qualitative and/or quantitative differences among fibroblast exosomes from various regions of the myocardium? and (5) Is it possible to alter exosomes to attenuate their detrimental effects and to enhance the benefits? It would be greatly beneficial to develop alternative strategies to engineer fibroblast (or any cell-specific) exosomes to enrich them with factors that target exosomes to the heart and appropriately repair the injury. Recent advancements in cardiovascular research indicate that exosomes may be used as a biomarker to determine heart disease at a much earlier stage than previously used biomarkers. As heart failure is a leading cause of morbidity and mortality both in developed and developing countries, developing novel biomarkers in the form of exosomes will meet a tremendous need to manage this number one lethal disease in a better manner.

## Author Contributions

PR generated the illustration. PR, RK, and SV wrote the manuscript. SV edited final draft of the manuscript. All authors drafted the manuscript.

## Conflict of Interest

The authors declare that the research was conducted in the absence of any commercial or financial relationships that could be construed as a potential conflict of interest.

## Abbreviations

α-SMA: α-smooth muscle actin
AP-1: activating protein-1
Azin1: antizyme inhibitor 1
CD: cluster of differentiation
CFs: cardiac fibroblasts
CM: cardiomyopathy
CVD: cardiovascular disease
DDR2: discoidin domain receptor 2
DNA: deoxyribonucleic acid
ECM: extracellular matrix
EPC: endothelial progenitor cell
EV: extracellular vesicle
EXO: exosome
Gal-3: Galectin-3
Hsp: heat-shock proteins
IGF1: insulin-like growth factor-1
IL-10: interleukin-10
IR: ischemia-reperfusion
Jnk1: c-JunN-terminal kinases 1
KO: knock out
lncRNAs: long non-coding RNAs
MAPKs: mitogen-activated protein kinases
MI: myocardial infarction
miR: Micro RNA
MMPs: matrix metalloproteinases
NcRNAs: non-coding RNAs
NF- κ B: nuclear factor κ B
NFAT: nuclear factor of activated T-cell
Nm: nano meter
PDGFs: platelet derived growth factors
PDLIM5: PDZ and LIM domain 5
PPAR- α: peroxisome proliferators-activated receptor alfa
RAS: renin-angiotensin system
RNAs: ribonucleic acids
ROS: reactive oxygen species
SDF-1: stromal-derived factor-1
SORBS2: sorbin and SH3 domain-containing protein 2
TGF- β: transforming growth factor- β
TIMPs: tissue inhibitors of metalloproteinases
TNF- α: tumor necrosis factor- α.
